# Effects of additives on the fermentation quality, *in vitro* digestibility and aerobic stability of mulberry (*Morus alba* L.) leaves silage

**DOI:** 10.5713/ajas.19.0420

**Published:** 2019-10-21

**Authors:** Zhihao Dong, Siran Wang, Jie Zhao, Junfeng Li, Tao Shao

**Affiliations:** 1 Institute of Ensiling and Processing of Grass, College of Agro-grassland Science, Nanjing Agricultural University, Nanjing 210095, China

**Keywords:** Mulberry Leaves, Additives, Fermentation Quality, Aerobic Stability, *In vitro* Digestibility

## Abstract

**Objective:**

To explore feed resources capable of replacing regular poor-quality fodder, this study was conducted to evaluate the effects of additives on the fermentation quality, *in vitro* digestibility and aerobic stability of mulberry leaves silage.

**Methods:**

The mulberry leaves were ensiled either untreated (control) or treated with 1×10^6^ cfu/g fresh matter *Lactobacillus plantarum* (L), 1% glucose (G), 3% molasses (M), a combination of 1% glucose and *Lactobacillus plantarum* (L+G), and a combination of 3% molasses and *Lactobacillus plantarum* (L+M). The fermentation quality and chemical composition were analyzed after 7, 14, 30, and 60 d, respectively. The 60-d silages were subjected to an aerobic stability test and fermented with buffered rumen fluid to measure the digestibility.

**Results:**

Inoculating lactic acid bacteria (LAB) resulted in more rapid increase in lactic acid concentrations and decline in pH of mulberry leaves silage as compared control. Higher acetic acid and lower ethanol and ammonia nitrogen concentrations (p<0.05) were observed in the LAB-inoculated silages as opposed to control during ensiling. The LAB-inoculated silages contained lower water-soluble carbohydrates compared with control during the first 14 d of ensiling, and lower neutral detergent fibre (p<0.05) concentrations as compared with non-LAB inoculated silages. Adding molasses alone increased (p<0.05) the digestibility of dry matter (DM). The aerobic stability of mulberry leaves silage was increased by LAB inoculation, whereas decreased by adding glucose or molasses.

**Conclusion:**

The LAB inoculation improved fermentation quality and aerobic stability of mulberry leaves silage, while adding glucose or molasses failed to affect the fermentation and impaired the aerobic stability. Inoculating LAB alone is recommended for mulberry leaves especially when ensiled at a relatively high DM.

## INTRODUCTION

The rapid development of China’s economy has triggered increasing demand for animal products from consumers. One challenge for further improvement of animal products has been the lack of adequate and high-quality green fodder. Exploring novel feed resources capable of replacing regular poor-quality fodder is important to alleviate this constraint.

Mulberry (*Morus alba* L.) originated from China and has been used widely in agriculture. They can grow well under various climate conditions ranging from temperate to tropic [1]. Mulberry fruit can be used in food and wine making. The leaves can be an ideal resource for animal feeding because of high biomass, crude protein content and digestibility [2]. In China, to wipe out poverty, the government has launched many projects encouraging the mulberry cultivation. Over the past a few years, mulberry production has been increasing dramatically and mulberry leaves has now become one of the major fodder resources. However, biomass harvest is seasonal with high accumulation in short time period. This results in a great need for identification of safe and efficient methods of storage to maintain the year-round supply. Based on economical and practical feasibility, ensiling might be the best option. This technique depends on lactic acid bacteria (LAB) fermenting soluble carbohydrates to organic acids, mainly lactic acid (LA), under anaerobic conditions. As a result, pH decreases and activities of undesirable microorganism are suppressed, leading to the conservation of dry matter (DM) and nutrients [3]. The success of ensiling depends on appropriate biological and chemical conditions that allow a rapid and sufficient decline in pH within silage. Under suboptimal conditions, silage additives are proposed to be used to manipulate fermentation, prolonging aerobic stability, and in some cases, improving animal performance [4].

Homofermentation LAB are the most common biological additives used in silage preservation. Previous studies observed that LAB inoculation successfully directed fermentation, resulting in reduced losses of DM and improved fermentation quality of silages [5–7]. Chemical additives, such as molasses and glucose, have been often used as fermentation stimulants to increase the amount of readily fermentable sugars for LAB. Li et al [8] showed that addition of molasses and glucose to king grass silage enhanced ensiling fermentation, resulting in rapid increase in LA content during the early stage of ensiling. However, possible beneficial effect of additives depends on the properties of the crops being ensiled. The use of these additives to modulate fermentation has not been always successful; some studies reported no benefits [9,10]. Therefore, systematical examination of the suitability of these additives for mulberry leaves silage is necessary, considering the importance of mulberry in agriculture.

The objective of this study was to investigate the effects of adding LAB, glucose and molasses on the fermentation characteristics, *in vitro* digestibility and aerobic stability of mulberry leaves silage.

## MATERIALS AND METHODS

### Ensilage material

Mulberry trees were cultivated in the experimental field of Nanjing Agricultural University (N31°14″, E118°22″, Nanjing, Jiangsu, China), which contained 10 plots (200 m^2^ per plot). Five plots of mulberry trees were randomly selected. On November, 2, 2016, the entire plant was harvested when reached approximately 120 cm, with a stubble of 20 cm above soil level. Leaves and stems were separated in a separator machine and the leaves were subsequently transported to laboratory. After wilting at room temperature (25°C) for 6 h, the mulberry leaves were chopped into a length of 2 to 3 cm with a forage chopper (F5, XiangLong, Co., Ltd., Linyi, China).

### Experiment design and silage preparation

Chopped mulberry leaves were divided into six groups. The six groups were randomly assigned to the following treatments: i) control (without additive); ii) L, LAB inoculant; iii) G, glucose; iv) M, molasses; v) L+G, a combination of L and G; or vi) L+M, a combination of L and M. The application rates of glucose and molasses were 1% and 3%, based on fresh matter (FM), respectively. The LAB inoculant used was supplied by a commercial company (Ecosyl Products Ltd., Stokesley, North Yorkshire, UK, mainly consisting of *Lactobacillus plantarum*). The application rate was 1.0×10^6^ colony forming units (cfu)/g of FM, according to the manufacturer’s specification. For the preparation of each silo, approximately 620 g of mulberry leaves were packed into a laboratory silo (polyethylene bottle, 1 L capacity) and manually compacted to a uniform density at 278 kg DM/m^3^. After being sealed with plastic caps and adhesive tapes, the silos were stored at ambient temperature (18°C to 22°C). Five replicates per treatment were randomly selected and opened after 7, 14, 30, and 60 d, respectively. The remaining silos were used for aerobic stability test after 60 d of ensiling.

### Chemical and microbial analyses

At silo opening, the silages were transferred to a plastic box for homogeneous mixing and divided into 2 subsamples. The first subsample was oven-dried at 60°C for 48 h to a constant weight to determine DM (934.01); the dried samples were analyzed for crude protein (CP, 984.13), neutral detergent fibre (NDF; with sodium sulfite and heat stable α-amylase, 2002.04) and acid detergent fibre (ADF, 973.18) with the method of Association of Official Analytical Chemists [11]. The water-soluble carbohydrates (WSC) were quantified as the method of Dong et al [12].

The second subsample (35 g) was extracted in 70 mL of deionized water at 4°C for 24 h to obtain the cold extract, which was used for analyzing silage fermentation parameters according to the procedure of Chen et al [13]. The pH of the water extract was measured with an electrode pH meter (HANNA pH 211, Hanna Instruments, Padova, Italy). The buffering capacity (BC) was determined with the method of Playne and McDonald [14]. Ammonia nitrogen (NH_3_-N) was measured using the method of Sun et al [15] and the value was expressed as g/kg of total nitrogen (TN). The concentrations of ethanol and organic acids including lactic, acetic (AA), propionic (PA), and butyric acid (BA) were quantified in the filtrates using high-performance liquid chromatography (HPLC) (Carbomix H-NP5 column, 55°C, 2.5 mM H_2_SO_4_, 0.5 mL/min).

The microbial populations were determined in the fresh mulberry leaves after ten-fold serially diluted to optimal ranges with sterilized saline solution (0.85% NaCl). The LAB was counted on de Man, Rogosa and Sharpe agar medium after incubation in an anaerobic incubator at 37°C for 3 d. Yeasts and aerobic bacteria were enumerated on potato dextrose agar and Agar medium, respectively, after incubation at 37°C for 2 d. All microbiological data were log10 transformed.

### *In vitro* digestibility measurements

Ground 60-d silage samples were put into filter bags (F57; ANKOM Technology, Macedon, NY, USA). Bags were heat-sealed and transported to 130-mL serum bottles. Rumen fluid was collected from four Boer male goats fed twice daily with a diet that contained 30% corn silage, 20% alfalfa silage, 40% corn grain and 10% soybean meal plus supplemental vitamins and minerals. The rumen fluid was strained through four layers of cheesecloth at 39°C under CO_2_ environment and then mixed with the 1:2 (v/v) buffer solution prepared as the description of Menke [16]. Sixty millilitres of the mixture was assigned to each serum bottle and subsequently incubated in a water bath at 39°C under CO_2_. After 72 h of incubation, samples were taken out from serum bottles and gently rinsed with cold tap water. Bags were weighed to determine *in vitro* DM digestibility (DM-D) after dried in oven at 65°C for 48 h. The *in vitro* NDF digestibility (NDF-D) were determined as losses in the NDF during the *in vitro* digestion process.

### Aerobic stability test

Mulberry leaves silage from each treatment was loosely filled into an open plastic bucket. A thermocouple wire was placed at the center of the silage biomass to record the temperature every 30 min for 7 d using a data logger (MDL-1048A; SMOWO Co., Ltd, Shanghai, China). Ambient temperature was recorded from a thermocouple wire in an empty bucket. To avoid contamination and allow air to penetrate the silage mass, the plastic bucket was covered with a double layer of cheesecloth. Aerobic stability was calculated as the number of hours before the temperature of the silage mass rose 2°C above ambient temperature.

### Statistical analysis

Statistical analysis was performed using the MIXED procedure of Statistical Analysis System (SAS Institute Inc., Cary, NC, USA). Silage chemical compositions were subjected to a two-way analysis of variance for a 5×4 (treatment×ensilage time) factorial arrangement using the following model: Y_ij_ = μ+S_i_+A_j_+S_i_×A_j_+ɛ_ij_ with μ the overall mean; S_i_ the effect of treatment; A_j_ the effect of ensilage time; S_i_×A_j_ the effect of the interaction between treatment and ensilage time; ɛ_ij_ the residual error. Tukey’s multiple comparison was used for the means separation. Significant differences were declared when p<0.05.

## RESULTS

### Characteristics of mulberry leaves

The chemical and microbial compositions of fresh mulberry leaves are shown in Table 1. The mulberry leaves had a DM of 38.5% FM. The CP, WSC, NDF, and ADF concentrations in mulberry leaves were 13.7%, 11.8%, 30.3%, and 15.7% DM, respectively. The BC was 223 mE/kg DM. The LAB, aerobic bacteria and yeasts counts were 6.73, 5.35, and 4.63 log cfu/g FM, respectively.

### Effects of additives on the fermentation quality

**Figure 1:**
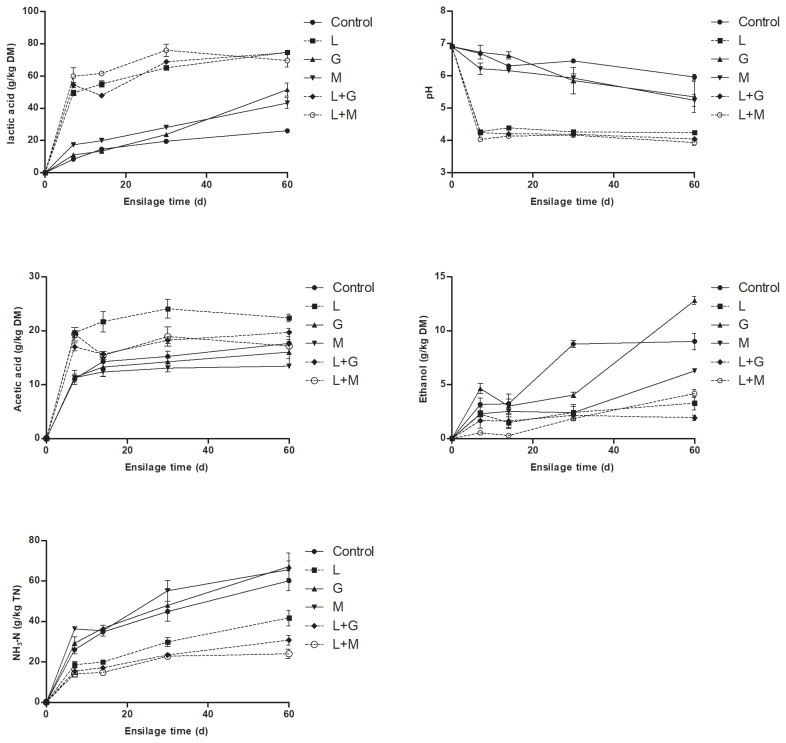
The dynamics of fermentation parameters of mulberry leaves during ensiling. DM, dry matter; NH_3_-N, ammonia nitrogen; TN, total nitrogen; L, *Lactobacillus plantarum*; G, glucose; M, molasses; L+G, *Lactobacillus plantarum* + glucose; L+M, *Lactobacillus plantarum* + molasses. The standard error of the means (n = 5) for the lactic acid, pH, acetic acid, ethanol and NH_3_-N/TN were 0.05, 0.006, 0.021, 0.015, and 0.061, respectively. Each of the effects of treatment, ensilage time and the interaction had a p-value of <0.05.

The dynamics of fermentation parameters during ensiling of mulberry leaves are shown in Figure 1. The LAB inoculation affected all fermentation parameters of mulberry leaves silage. Apparently higher (p<0.05) LA concentrations in L, L+G, and L+M silages were observed as opposed to control during ensiling. However, G and M treatment had little effect on LA production. Corresponding with the increases in LA concentrations, pH in L, L+G, and L+M silages dropped rapidly to <4.30 by d 7, whereas pH in control, G and M silages maintained >5.20 until the end of ensiling. During ensiling AA concentration was always observed to be highest in L silage, whereas lowest in M silage. The ethanol and NH_3_-N accumulated gradually in all the silages with increasing time of ensiling. LAB-inoculated silages (L, L+G, and L+M silage) contained numerically or significantly lower ethanol concentrations as opposed to control, G and M silage during ensiling. Inoculating LAB distinctly depressed the production of NH_3_-N, as indicated by the lower (p<0.05) NH_3_-N concentrations in the L, L+G, and L+M silages as opposed to the control, G and M silages during ensiling.

**Figure 2:**
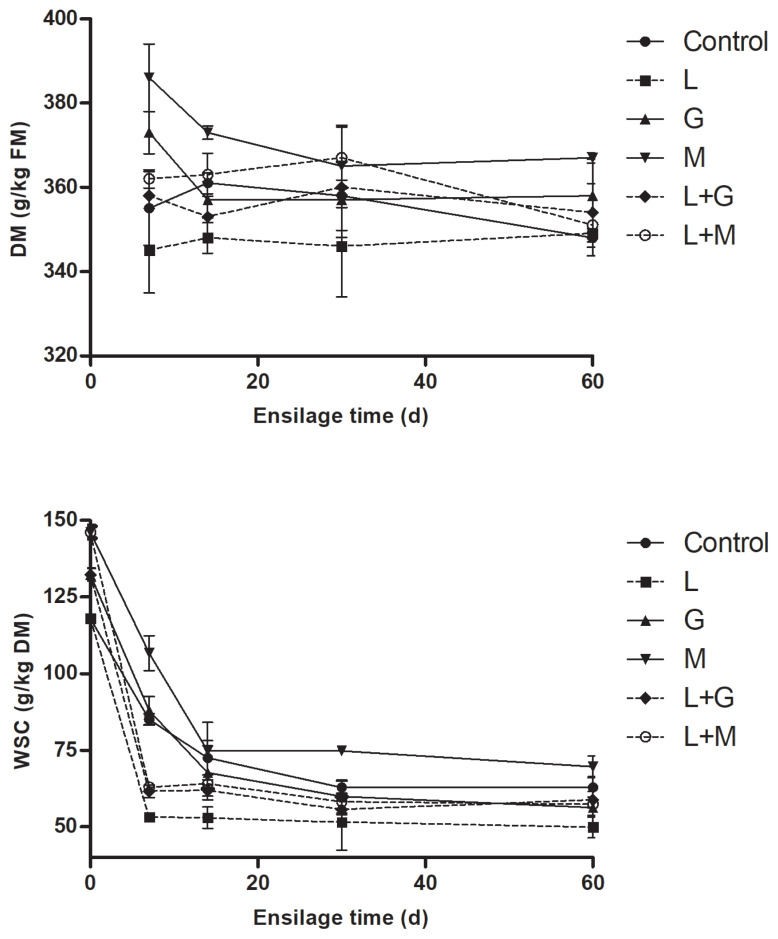
The dynamics of dry matter (DM) and water-soluble carbohydrates (WSC) of mulberry leaves during ensiling. L, *Lactobacillus plantarum*; G, glucose; M, molasses; L+G, *Lactobacillus plantarum* + glucose; L+M, *Lactobacillus plantarum* + molasses; FM, fresh matter. The standard error of the means (n = 5) for the DM and WSC were 0.35 and 0.09, respectively. Each of the effects of treatment, ensilage time and the interaction had a p-value of <0.05.

Dynamics of DM and WSC concentrations of mulberry leaves during ensiling are given in Figure 2. The DM and WSC tended (p>0.05) to decrease in all the silages with prolonged time of ensiling. At most intervals of ensiling, DM concentrations was observed to be highest in M silage, whereas lowest in L silage. Compared with non-LAB inoculated silages (control, G and M silage), more rapid and larger extent of WSC reductions were observed in LAB-inoculated silages (L, L+G, and L+M silage) during the first 14 d of ensiling. The WSC concentration was lowest in L silage and highest in M silage during ensiling.

### Effects of additives on the chemical compositions and *in vitro* digestibility

The effects of additives on the CP, NDF, ADF, and *in vitro* digestibility of mulberry leaves ensiled for 60 d are displayed in Table 2. The CP concentration was not affected by the treatments. Lower (p<0.05) NDF concentrations in L, L+G, and L+M silages were observed compared with those in control, G and M silages. Compared with non-LAB inculcated silages, the reduction in ADF contents was not significant by LAB inculcation alone, whereas became significant (p<0.05) when combined with G or M. Adding M alone increased the DM-D, however the NDF-D were not significantly influenced by the treatments.

### Effects of additives on the aerobic stability

**Figure 3:**
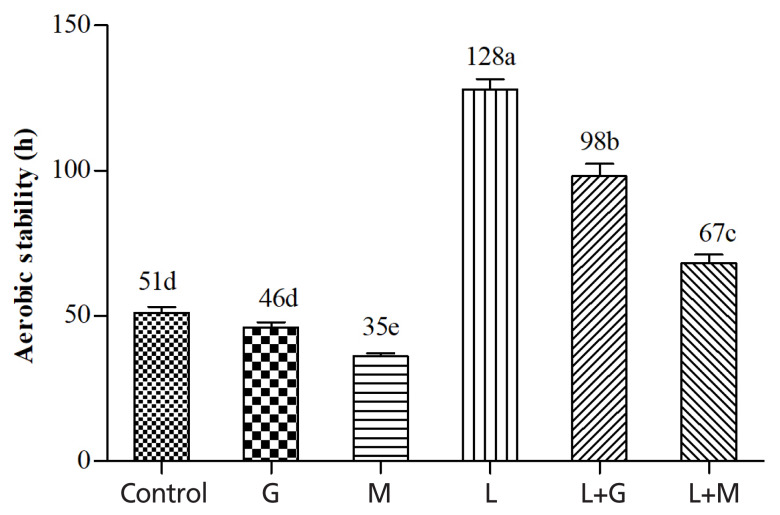
Aerobic stability of mulberry leaves silage. Vertical bars are the standard errors of the means, bars with different letters differ (p<0.05, n = 5). Control, without additive; G, glucose; M, molasses; L, *Lactobacillus plantarum*; L+G, *Lactobacillus plantarum* + glucose; L+M, *Lactobacillus plantarum* + molasses

Figure 3 shows the effects of additives on aerobic stability of mulberry leaves silage. The control kept aerobically stable for 51 h. The G and M silages began to spoil after 46 h and 35 h, respectively. The L, L+G, and L+M treatments increased the aerobic stability to 128, 98, and 67 h, respectively.

## DISCUSSION

The epiphytic LAB numbers and WSC content have become significant factors in predicting the adequacy of silage fermentation and determining whether or not to apply additives to ensilage materials [17]. Chen et al [3] reported the minimum number of epiphytic LAB required for the achievement of quality fermentation is 5 log_10_ cfu/g FM and the content of WSC is 7.0% DM. However, other important factors, such as DM content and the epiphytic LAB compositions, could also affect the resultant fermentation [10]. Considering these, identification of the effects of additives on the fermentation quality of mulberry leaves is still necessary despite containing a sufficient number of LAB (6.73 log_10_ cfu/g FM) and adequate WSC concentration (11.8% DM).

After 60 d of ensiling high pH (5.96) and low LA concentration (2.61% DM) indicated poor fermentation quality of the control silage. Despite more fermentable substrate provided by molasses and glucose addition, these treatments failed to induce intense LA fermentation suggesting that fermentable sugar was not the limiting factor for improved fermentation quality of mulberry leaves silage. In contrast, inoculation with *Lactobacillus plantarum* triggered vigorous LA fermentation. Based on the research of Meeske et al [18], when the DM content of an ensiled crop reaches 32% FM, a pH of 4.53 can be the criterion for effective preservation. Therefore, the low terminal pH (<4.30) of the LAB-inoculated silages indicated satisfactory fermentation qualities. However, opposite results were obtained by Zhang et al [9], who reported that sufficient number of epiphytic LAB resulted in no effect of LAB inoculation on the fermentation quality of mulberry leaves. A possible explanation to this discrepancy could be the difference in DM content of mulberry leaves between two studies. Whiter and Kung [19] found that only 10% of the total population of epiphytic LAB on forages could grow on a modified agar with a water activity of 0.952 (corresponding to forage with a DM content of 50%), while colonies of *Lactobacillus plantarum* were detectable even when the water activity of the media was 0.949. These observations suggested that, compared with epiphytic LAB, *Lactobacillus plantarum* has a higher ability to thrive in high DM silage. In the experiment, despite mulberry leaves being wilted for only 6 h, a relatively higher DM (38.5% vs 21.5% DM) content was obtained as compared with that in study of Zhang et al [9]. This high DM may depress the activity of epiphytic LAB, which explained the poor fermentation quality of control silage. Inoculation of *Lactobacillus plantarum* to mulberry leaves provided sufficient number of LAB with high osmotolerant ability; thus, it could be expected to improve the fermentation quality.

The *Lactobacillus plantarum*, used in the experiment, belongs to *lactobacilli* and is capable of quickly producing large amounts of LA by fermenting a wide variety of substrates. In fact, the *Lactobacillus plantarum* is a facultative homofermentation species. They can adapt to diverse conditions by altering metabolisms. Under normal conditions of high sugar content and limited access to oxygen, pyruvate is reduced to LA, a metabolic pathway known as homolactic fermentation. However, when available sugars become limited, *Lactobacillus plantarum* can alter its metabolism to pentose phosphate pathway, which is characterized by initial dehydrogenation, followed by decarboxylation leading to significant amounts of other end products, including CO_2_, ethanol, or AA [20]. In the present study, rapid depletion of WSC may result in the alteration of sugar metabolism of *Lactobacillus plantarum*, explaining the highest AA concentration of L silage after 7 d of ensiling.

Ethanol can be produced from glucose by yeasts and enterobacteria. Lower ethanol content in LAB-inoculated silages as opposed to non-inoculated LAB silages might be ascribed to the restriction of the ethanol-producing microorganisms by rapid pH decline. These results were similarly obtained by Cao et al [21], who reported that inoculation with homofermentative LAB improved the fermentation by increasing LA concentration and decreasing pH values, which inhibited the growth of undesirable bacteria and consequently reduced ethanol concentration.

The NH_3_-N is indicative of proteolytic activity and amino acid deamination and decarboxylation, which typically reduce the nutritive value of silages. The satisfactory ranges of NH_3_-N (<10% TN) within all the silages indicated no extensive proteolysis occurred. This low extent of protein degradation was possibly associated with the depression of protease activity resulting from the high DM. It was observed that NH_3_-N concentrations in LAB-inoculated silages were reduced in contrast to non-LAB inoculated silages and this might be attributed to rapid decline of pH that suppressed deamination and decarboxylation of amino acids. Similarly, Guo et al [22] reported that acidifying alfalfa with formic acid resulted in lower NH_3_-N concentrations during the early stage of ensiling compared with control. In the present experiment, the BA and PA concentrations in all the silages were below the detection limit, indicating that clostridia and other undesirable organisms did not develope in large numbers.

Due to the molasses addition, M silage exhibited highest DM during ensiling. During the first 7 d of ensiling WSC concentration in LAB-inoculated silages were apparently lower compared with non-LAB inoculated silages. The decrease in WSC concentrations coincided with the increase in LA concentrations, suggesting that WSC was largely consumed by *Lactobacillus plantarum* for LA production. These results were in accordance with Filya et al [23], who found WSC remaining in alfalfa silage were significantly higher in non-inoculated control than those in the LAB-inoculated counterpart after ensiling. During ensiling M silage showed highest WSC concentration, which might be attributed to the weak conversion efficiency of WSC by epiphytic LAB resulting in more WSC being retained in the silage [24].

After 60 d of ensiling, lower NDF contents in the LAB-inoculated silages were observed in comparison to those in non-LAB inoculated silages. This was likely owing to acid hydrolysis of more digestible cell wall fractions during ensiling. It has been found that arabinose, a key component involved in crossing linking between lignin and arabinoxylans, is sensitive to acid hydrolysis [25]. Solubilization of arabinose may alter the degradability of cell wall. Lowest ADF contents in L+G and L+M silage among all the silages could be a combined consequence of acid hydrolysis and reduced relative contents of structural carbohydrates due to more WSC retained in resultant silages.

Digestibility parameters of forages are considered to be essential values in estimation of their nutritive value to ruminants. The digestibility of silage is influenced by the amount of fermentable sugar and CP available for rumen microbial degradation during *in vitro* incubation [26]. Sahoo and Walli [27] previously found that molasses addition improved the digestibility of nutrients as a result of a more conducive environment for rumen microbes. Higher DM-D of M silage than other silages may be explained by the higher residual WSC content. In the experiment, the *in vitro* digestibility of NDF was unaffected by the treatments. Weinberg et al [28] evaluated the effect of LAB inoculants on the NDF-D, and found none of these bacteria improved NDF-D. In that work, they stated that possibly due to some solubilization of the hemicellulose during ensiling the digestibility of residual NDF was not changed or even decreased.

Aerobic stability is of great importance because it is not only a potential cause of nutrient and DM losses, it also leads to health risks to animals and humans due to mycotoxins produced by undesirable microorganisms. In the experiment, high DM restricted the extent of fermentation, resulting in high concentration of residual WSC in the resultant silage, which was a potential source of readily available substrate for the growth of aerobic microflora when the silages are exposed to air. This may explain the poor aerobic stability of the control silage. Likewise, M treatment increased the WSC concentration retained in the resultant silage, which was probably responsible for the decreased aerobic stability compared with control. In contrast, LAB inoculation reduced residual WSC concentrations, contributing to the increased aerobic stability. Furthermore, it is well known that AA is one of the most effective substances for inhibition of spoilage microorganisms [29]. Increased accumulation of AA in the LAB-inoculated silages may be also contributable for the increased aerobic stability.

## CONCLUSION

The LAB inoculation improved fermentation quality and aerobic stability of mulberry leaves silage, while adding glucose or molasses failed to affect the fermentation and impaired the aerobic stability. Based on the results obtained in the experiment, inoculating LAB alone is advisable especially when mulberry leaves are ensiled at a relatively high DM.

## Figures and Tables

**Table 1:** Chemical (% DM, unless stated otherwise) and microbial compositions (on fresh matter basis) of mulberry leaves

Items	Mulberry leaves
Chemical composition	
DM (% fresh matter)	38.5
CP	13.7
WSC	11.8
BC (mE/kg DM)	223
NDF	30.3
ADF	15.7
Microbial populations	
LAB (log cfu/g)	6.73
Aerobic bacteria (log cfu/g)	5.35
Yeasts (log cfu/g)	4.63

DM, dry matter; CP, crude protein; WSC, water soluble carbohydrates; BC, buffer capacity; NDF, neutral detergent fiber; ADF; acid detergent fiber; LAB, lactic acid bacteria; cfu, colony-forming units.

**Table 2:** Chemical compositions and in vitro digestibility of mulberry leaves silage

Items	Control	L1)	G1)	M1)	L+G1)	L+M1)	SEM	p-value
Chemical compositions (g/kg DM)								
CP	14.8	14.2	14.4	13.6	13.7	14.9	0.201	0.546
NDF	26.1^A^	23.5^B^	25.8^A^	26.4^A^	23.0B^C^	21.9^C^	0.456	0.013
ADF	15.2^A^	14.6^A^	14.3^A^	14.3^A^	13.7^B^	13.0^B^	0.311	0.045
Digestibility (%)								
DM-D	43.3^B^	41.2^B^	41.6^B^	53.6^A^	42.8^B^	44.3^B^	0.860	0.040
NDF-D	34.1	31.8	33.6	35.4	32.9	33.0	0.680	0.106

SEM, standard error of means (n = 5); DM, dry matter; CP, crude protein; NDF, neutral detergent fibre; ADF, acid detergent fibre; DM-D, in vitro dry matter digestibility; NDF-D, *in vitro* neutral detergent fibre digestibility.

1) L, *Lactobacillus plantarum*; G, glucose; M, molasses; L+G, *Lactobacillus plantarum* + glucose; L+M, *Lactobacillus plantarum* + molasses.

A–C Means in the same row with different letters differed significantly (p<0.05, n = 5).
